# D-Dimer as Biomarker for Early Prediction of Clinical Outcomes in Patients With Severe Invasive Infections Due to *Streptococcus Pneumoniae* and *Neisseria Meningitidis*

**DOI:** 10.3389/fmed.2021.627830

**Published:** 2021-04-15

**Authors:** Simone Meini, Emanuela Sozio, Giacomo Bertolino, Francesco Sbrana, Andrea Ripoli, Carlo Pallotto, Bruno Viaggi, Roberto Andreini, Vittorio Attanasio, Carolina Rescigno, Luigi Atripaldi, Silvia Leonardi, Mariano Bernardo, Carlo Tascini

**Affiliations:** ^1^Internal Medicine Unit, Felice Lotti Hospital of Pontedera, Azienda Unità Sanitaria Locale Toscana Nord-Ovest, Pisa, Italy; ^2^Infectious Disease Unit, Department of Medicine, University of Udine, Udine, Italy; ^3^Pharmaceutical Department, Ospedale di Sassuolo, Modena, Italy; ^4^Fondazione Toscana Gabriele Monasterio, Pisa, Italy; ^5^Infectious Diseases Unit 1, Santa Maria Annunziata Hospital, Azienda Unità Sanitaria Locale Toscana Centro, Florence, Italy; ^6^Section of Infectious Diseases, Department of Medicine, University of Perugia, Perugia, Italy; ^7^Neuro Intensive Care Unit, Department of Anesthesiology, Careggi University Hospital, Florence, Italy; ^8^First Division of Infectious Diseases, Cotugno Hospital, Azienda Ospedaliera dei Colli, Naples, Italy; ^9^Central Laboratory, Azienda Ospedaliera dei Colli, Naples, Italy

**Keywords:** D-dimer, biomarker, sepsis, meningitis, *Streptococcus pneumoniae*, *Neisseria meningitidis*, mortality

## Abstract

Sepsis is defined as life-threatening organ dysfunction caused by a dysregulated host response to infection; no current clinical measure adequately reflects the concept of dysregulated response. Coagulation plays a pivotal role in the normal response to pathogens (immunothrombosis), thus the evolution toward sepsis-induced coagulopathy could be individuate through coagulation/fibrinolysis-related biomarkers. We focused on the role of D-dimer assessed within 24 h after admission in predicting clinical outcomes in a cohort of 270 patients hospitalized in a 79 months period for meningitis and/or bloodstream infections due to *Streptococcus pneumoniae* (*n* = 162) or *Neisseria meningitidis* (*n* = 108). Comparisons were performed with unpaired *t*-test, Mann-Whitney-test or chi-squared-test with continuity correction, as appropriate, and multivariable logistic regression analysis was performed with Bayesian model averaging. In-hospital mortality was 14.8% for the overall population, significantly higher in *S. pneumoniae* than in *N. meningitidis* patients: 19.1 vs. 8.3%, respectively (*p* = 0.014). At univariable logistic regression analysis the following variables were significantly associated with in-hospital mortality: pneumococcal etiology, female sex, age, ICU admission, SOFA score, septic shock, MODS, and D-dimer levels. At multivariable analysis D-dimer showed an effect only in *N. meningitidis* subgroup: as 500 ng/mL of D-dimer increased, the probability of unfavorable outcome increased on average by 4%. Median D-dimer was significantly higher in *N. meningitidis* than in *S. pneumoniae* patients (1,314 vs. 1,055 ng/mL, *p* = 0.009). For *N. meningitidis* in-hospital mortality was 0% for D-dimer <500 ng/mL, very low (3.5%) for D-dimer <7,000 ng/mL, and increased to 26.1% for D-dimer >7,000 ng/mL. Kaplan-Meier analysis of in-hospital mortality showed for *N. meningitidis* infections a statistically significant difference for D-dimer >7,000 ng/mL compared to values <500 ng/mL (*p* = 0.021) and 500–3,000 ng/mL (*p* = 0.002). For *S. pneumoniae* the mortality risk resulted always high, over 10%, irrespective by D-dimer values. In conclusion, D-dimer is rapid to be obtained, at low cost and available everywhere, and can help stratify the risk of in-hospital mortality and complications in patients with invasive infections due to *N. meningitidis*: D-dimer <500 ng/mL excludes any further complications, and a cut-off of 7,000 ng/mL seems able to predict a significantly increased mortality risk from much <10% to over 25%.

## Introduction

Sepsis is a heterogeneous syndrome defined as a life-threatening organ dysfunction caused by a dysregulated host response to infection, associated with an in-hospital mortality >10% (1, 2). The role of biomarkers [biological observations that substitute for and ideally predict a clinically relevant endpoint (3)] for diagnostic and prognostic assessment in case of sepsis has been extensively investigated in literature, but to date no current clinical measure seems able to reflect the concept of “dysregulated host response” (1); therefore, identifying a biomarker reflective of host-response interaction would be of great interest.

It has been known for several years that coagulation plays a pivotal role in the physiological host response to pathogens: immunothrombosis, mediated by immune cells and by specific thrombosis-related molecules, leads to the generation of a localized intravascular scaffold that facilitates the recognition, containment and killing of pathogens, limiting their diffusion through the circulatory system, thus protecting host integrity and limiting major organ damage (4): the loss of the physiologically localized activation of coagulation, the signature feature of disseminated intravascular coagulation (DIC), is a hallmark of sepsis and invasive infections (4). In general, during a systemic infection, both extrinsic and contact coagulation pathways are activated, the first mainly triggered by the intravascular tissue factor (TF) expressed on the monocytes and extracellular microparticles (MPs) surfaces, the second by the cell-free DNA associated with neutrophil extracellular traps (NETs) and by the phosphatidylserine residues present in various cell membranes (4). Sepsis-associated coagulopathy is characterized by concomitant activation of coagulation, down-regulation of physiological anticoagulants, and inhibition of fibrinolysis (5), finally leading to the generation of a variable amount of fibrin-related markers, such as the D-dimer.

Several biomarkers indicating a strong fibrinolytic shutdown, including low D-dimer levels, were found to be associated with a reduced survival in patients with sepsis (6); recently a very high mortality among the few sepsis patients having normal D-dimer levels (<500 ng/mL) was once again reported (7). On the contrary, other studies showed that the mortality is effectively predicted by high levels, with D-dimer ≥4,200 ng/mL effective in identifying the patients with infective endocarditis (IE) (8) and ≥4,000 ng/mL for those with bloodstream infections (BSI) (9); moreover, a linear relationship between high D-dimer levels and mortality in sepsis patients admitted to the emergency department has been reported (10). On-admission D-dimer levels ≥2,000 ng/mL were able to effectively predict in-hospital mortality also among Covid-19 patients (11).

D-dimer seems to be therefore an interesting biomarker in predicting prognosis in different models of infection, able to reflect the synthesis of the complex balance between the pro- and anti-thrombotic and the pro- and anti-fibrinolytic drive occurring during sepsis-related coagulopathy. However, the dysregulated host response occurring during an invasive infection depends on the specific pathogen involved (4, 12); it is plausible to hypothesize that the levels of a coagulative biomarker, and the related thresholds, should be assessed and differently interpreted for each pathogen. Infections due to *Streptococcus pneumoniae* and *Neisseria meningitidis* represent two excellent models of severe invasive infections due, respectively, to Gram-positive and Gram-negative cocci whose pathogenesis is usually characterized by an extensive coagulopathy and sometimes overt-DIC.

The aim of this study is to evaluate in a large cohort of patients with meningitis and/or BSI due to *S. pneumoniae* or *N. meningitidis* the main clinical and laboratory characteristics and variables associated with in-hospital mortality and complications, with a particular focus on the role of D-dimer as biomarker for early prediction of clinically relevant outcomes.

## Materials and Methods

### Study Design, Setting, and Subjects

A retrospective study was conducted from November 2012 to May 2019 (79 months), enrolling all consecutive patients admitted for meningitis and/or BSI due to *S. pneumoniae* or *N. meningitidis* to the First Division of Infectious Diseases (FDID) of Cotugno Hospital, in Naples, Italy, a referral tertiary hospital covering an area (Campania region) with 5,800,000 inhabitants. This division has 25 beds dedicated to the management of invasive bacterial infections and other infectious diseases.

All patients enrolled in this study underwent blood cultures and, if not contraindicated, lumbar puncture.

In each patient, a complete blood analysis, including assessment of D-dimer, was performed within the first 24 h after admission. Patients for which the main laboratory and clinical data and primary clinical outcome were available were enrolled for the final analysis. No exclusion criteria were applied regarding the age of patients and the severity of clinical condition.

We assessed in each patient the presence of purpura with onset within 24 h after admission.

In each patient, within the first 24 h after admission, the ISTH overt-DIC score (13, 14), the SIC score (15), the Sequential Organ Failure Assessment (SOFA) score, were calculated according to current definitions, and multiple organ dysfunction syndrome (MODS) and septic shock (1) status was assessed.

### Data Collection

The computerized database of the FDID was searched to identify all consecutive inpatients with meningitis and/or BSI due to *S. pneumoniae* or *N. meningitidis*, diagnosed between November 2012 and May 2019.

Data were obtained by electronic clinical chart review and interrogation of laboratory information systems and collected in a dedicated case record form.

Clinical information, including demographics, antimicrobial therapy, and laboratory data were retrieved from medical charts.

Clinical outcomes were retrieved from the electronic clinical chart related to the index hospitalization.

This study was exempt from institutional review board oversight because of its retrospective nature and the anonymity of pooled data.

### Definitions

Sepsis and septic shock were defined according to the sepsis-3 definition (1).

Multiple organ dysfunction syndrome (MODS) was defined as the presence of altered function involving at least two organ systems in an acutely ill patient such that homeostasis cannot be maintained without intervention.

Purpura was defined as petechial rash rapidly spreading in extent and depth, evolving into skin necrosis.

Antibiotic therapy was defined as adequate if the isolated pathogen resulted susceptible to the tested antibiotic according to EUCAST clinical breakpoints effective during the study period.

### Clinical Outcomes

The primary outcome was in-hospital mortality.

The secondary outcome was a composite of in-hospital mortality, amputations, hearing loss, neurological complications (stroke, transitory ischemic attack, brain abscess, epilepsy).

### Coagulation Testing

Prothrombin time/INR, fibrinogen and D-dimer were measured using the ACL TOP analyzer (Instrumentation Laboratory-IL, Werfen Group).

Q.F.A. Thrombin (Bovine) kit was used for the quantitative determination of fibrinogen, based on the Clauss method, in human citrated plasma on the IL Coagulation Systems.

The quantitative determination of D-dimer in human citrated plasma was determined by the automated latex enhanced immunoassay HemosIL D-Dimer HS (0020007700).

D-dimer classes were divided as follows: <500, 500–3,000, 3,000–7,000, and >7,000 ng/mL. The cut-off values of 3,000 and 7,000 ng/mL were chosen according to the proposal of ISTH about the proper values for moderate (2 points) and severe (3 points) increase in the ISTH overt-DIC score (14).

### Microbiology Laboratory Methods

Blood cultures were inoculated in BD BACTEC^TM^ blood culture bottles (Becton, Dickinson and Company, NJ, U.S.) and monitored using BD BACTEC^TM^ FX instrument for up to 5 days. Positive cultures were sub-cultured and identified to the species level by Vitek®MS or Vitek®2 (BioMérieux; Marcy-l'Étoile, France).

Cerebrospinal fluid (CSF) was first examined by a Gram-stained smear, and the appropriate culture media was inoculated. Blood agar and chocolate agar plates should be incubated at 35°C in an atmosphere enriched with carbon dioxide. CSF was cultured and identified to the species level by Vitek®MS or Vitek®2 (BioMérieux; Marcy-l'Étoile, France).

Susceptibility testing was performed using Vitek®2 system and interpreted according to EUCAST criteria effective during the study period.

Polymerase chain reaction (PCR) for the detection of meningococcal and pneumococcal DNA on blood specimens, antigen detection on CSF and Biofire® FilmArray^TM^ meningitis/encephalitis (ME) panel (a multiplex PCR assay which can detect the most commonly identified pathogens in central nervous system infections) were performed when adequate and/or available during the study period.

### Statistical Analysis

Descriptive analysis of the data was carried out using mean values and standard deviation (SD) or median values and interquartile range (IQR) for the quantitative variables, as appropriate, and percentage values for the qualitative ones. Normality of the variables was assessed with the Shapiro-Wilk-test. Comparisons between groups were performed with unpaired two-tailed *t*-test, Mann-Whitney-test or chi-squared-test with continuity correction, as appropriate.

The data related to in-hospital mortality was analyzed with Kaplan-Meier curves.

The association with the outcome of each considered predictor was investigated with univariable logistic regression. Predictors with a *p*-value <0.20 at the univariable logistic regression were considered for multivariable analysis.

The variable “Interaction” is “D-dimer-*N. meningitidis*,” and represents the D-dimer in this specific subgroup; this explicitly entered the analysis because it expresses the main hypothesis of the work. For the D-dimer odds ratio (OR), an increase of 500 ng/mL was considered.

Multivariable logistic regression analysis was performed with Bayesian model averaging (BMA) (16) to address model uncertainty, producing a posterior probability for each possible model and predictor. As a result of multivariable analysis, in addition to OR, the probability that the single variable has a non-zero effect in the final multivariable model [posterior probability, *p* (*b* ≠ 0)] was reported. The variables with *p* (*b* ≠ 0) > 0.80 were selected.

A *p*-value of <0.05 was considered statistically significant.

Analyses were performed using the *R* open-source statistical software and the SPSS statistical package (version 23 for Windows. SPSS, Inc. Chicago, Ill).

## Results

### Patients' General Characteristics

During the 79 months study period, there have been 3,130 admissions in the FDID of Cotugno Hospital. We retrospectively included in the final analysis 162 patients with invasive infections due to *S. pneumoniae* (male: 48.8%; mean age: 45 years, SD 26; range: 0–89 years), and 108 patients with invasive infections due to *N. meningitidis* (male: 55.6%; mean age: 22 years, SD 22, range: 0–90 years), for a total of 270 cases. Only 3 patients out of 273 cases initially retrieved were excluded, 2 because missing data about D-dimer levels and 1 for incomplete data about several clinical characteristics.

Median age was significantly higher in *S. pneumoniae* than in *N. meningitidis* patients (53.5 years, IQR 21–67, vs. 15 years, IQR 3–39, *p* < 0.001).

Regarding 162 invasive infections due to *S. pneumoniae*, 132 patients had meningitis (86/132, 65.2%, with concomitant BSI), and 30 had isolated BSI. Regarding 108 invasive infections due to *N. meningitidis*, 90 patients had meningitis (66/90, 73.3%, with concomitant BSI), and 18 had isolated BSI.

Table 1 shows the results of the analysis of variables for different etiologies.

**Table 1:** Analysis of variables for different etiologies.

		***S. pneumoniae* (*n* = 162)**	***N. meningitidis* (*n* = 108)**	***p*-value**
Males, *n* (%)		79 (48.8%)	60 (55.6%)	0.274
Age (years), median (IQR)		53.5 (21.0–67.0)	15.0 (3.0–39.0)	**<0.001**
SOFA, median (IQR)		3.0 (2.2–6.0)	3.0 (2.0–5.0)	0.465
PLT (×10^9^/mm^3^), median (IQR)		195.5 (145.0–260.0)	167.0 (129.0–232.0)	**0.019**
Purpura, *n* (%)		14 (8.6%)	62 (57.4%)	**<0.001**
Splenectomy, *n* (%)		11 (6.8%)	1 (0.9%)	**0.031**
BSI isolated, *n* (%)		30 (18.5%)	18 (16.7%)	0.776
Meningitis isolated, *n* (%)		46 (28.6%)	24 (22.2%)	0.245
Meningitis + BSI, *n* (%)		86 (53.1%)	66 (61.1%)	0.122
D-dimer classes (ng/mL)	<500	35 (21.6%)	18 (16.7%)	**0.002**
	500–3,000	101 (62.3%)	52 (48.1%)	
	3,000–7,000	15 (9.3%)	15 (13.9%)	
	>7,000	11 (6.8%)	23 (21.3%)	
D-Dimer >3,000 ng/mL, *n* (%)		136 (84.0%)	70 (64.8%)	**<0.001**
D-Dimer >7,000 ng/mL, *n* (%)		11 (6.8%)	23 (21.3%)	**<0.001**
Fibrinogen (mg/dL), median (IQR)		619.5 (469.0–777.0)	574.5 (427.0–720.0)	0.114
D-dimer (ng/mL), median (IQR)		1055.0 (585.0–2239.0)	1314.0 (706.0–4223.0)	**0.009**
ISTH score positive, *n* (%)		4 (2.7%)	20 (20.6%)	**<0.001**
mISTH score positive, *n* (%)		13 (8.0%)	34 (32.1%)	**<0.001**
SIC score positive, *n* (%)		61 (37.9%)	63 (58.9%)	**0.001**
ISTH score, median (IQR)		1.0 (1.0–2.0)	2.0 (1.0–4.0)	**<0.001**
mISTH score, median (IQR)		1.0 (1.0–2.0)	2.0 (1.0–4.0)	**<0.001**
SIC score, median (IQR)		3.0 (2.0–4.0)	4.0 (3.0–4.0)	**0.003**
In-hospital mortality, *n* (%)		31 (19.1%)	9 (8.3%)	**0.014**
Composite of mortality or complications, *n* (%)		75 (46.3%)	20 (18.5%)	**<0.001**
Septic shock, *n* (%)		48 (29.6%)	40 (37.0%)	0.229
MODS, *n* (%)		57 (35.2%)	28 (25.9%)	0.101
ICU admission, *n* (%)		78 (48.1%)	65 (60.2%)	0.052

*In all patients (pts), the ISTH overt-DIC score and the SIC score were calculated according to definitions (13–15) within the first 24 h after admission. The cut-off of ISTH overt-DIC score is 5 (13). The cut-off of modified ISTH (mISTH) overt-DIC score (without fibrinogen) is 4 (17). The cut-off of SIC score is 4 (15). Bold text indicates statistically significant difference (p < 0.05)*.

All patients (100%) received an adequate empiric antibiotic treatment before blood and CSF culture results and then an adequate targeted therapy; 96.7% were treated with corticosteroids.

The median in-hospital length of stay of overall population was 17 days (range 0–129 days).

143/270 (53%) patients needed admission in intensive care unit (ICU) during the hospital stay.

### SOFA Score

48/270 patients (17.8%) had SOFA score <2 (absence of criteria for sepsis diagnosis): none of these died. No statistically significant difference (*p* = 0.465) was found for median SOFA scores between *S. pneumoniae* (3, IQR 2–6) and *N. meningitidis* (3, IQR 2–5) groups (Table 1).

For *S. pneumoniae* infections, the median SOFA was 7 (IQR 4–8) in non-survivors vs. 3 (IQR 2–5) in survivors (*p* < 0.001; **Table 3**), while for *N. meningitidis* infections it was 6.5 (IQR 4–9.5) vs. 3 (IQR 2–5) (*p* = 0.010; **Table 4**).

At the univariable and multivariable logistic regression analysis (Table 2), the SOFA score was significantly associated with in-hospital mortality in the overall population.

**Table 2:** Univariable and multivariable logistic regression analysis (Bayesian model averaging).

	**Univariable logistic regression**		**Bayesian model averaging**	
	**OR (95% CI)**	***p*-value**	**OR (95% CI)**	***p* (*b* ≠ 0)**
*N. meningitidis*-*S. pneumoniae*	0.250 (0.133–0.452)	<0.001	0.117 (0.111–0.124)	1.000
Fibrinogen	0.999 (0.998–1.000)	0.483	–	–
D-dimer (×500 ng/mL)	1.025 (1.007–1.047)	0.010	1.004 (1.003–1.006)	0.157
Male sex	0.533 (0.312–0.904)	0.020	0.886 (0.855–0.918)	0.206
Age	1.022 (1.011–1.033)	<0.001	1.001 (1.000–1.002)	0.071
ICU admission	2.587 (1.499–4.549)	<0.001	1.241 (1.175–1.310)	0.254
SOFA	1.221 (1.118–1.342)	<0.001	1.185 (1.174–1.956)	0.926
Platelet count	1.001 (0.998–1.003)	0.686	–	–
Splenectomy	0.968 (0.247–3.302)	0.959	–	–
Septic shock	2.808 (1.621–4.907)	<0.001	1.583 (1.486–1.687)	0.533
MODS	3.519 (2.012–6.234)	<0.001	1.003 (0.992–1.015)	0.030
Purpura	0.705 (0.385–1.261)	0.245	–	–
BSI isolated	0.628 (0.293–1.274)	0.211	–	–
Meningitis isolated	0.862 (0.458–1.587)	0.638	–	–
Meningitis + BSI	1.581 (0.927–2.725)	0.095	1.003 (0.996–1.010)	0.025
ISTH score	1.034 (0.882–1.208)	0.678	–	–
SIC score	1.224 (0.971–1.559)	0.092	1.010 (1.003–1.017)	0.054
Interaction (×500 D-dimer)	1.014 (0.996–1.027)	0.140	1.041 (1.038–1.045)	0.849

*The variables that are selected in the final multivariable model are N. meningitidis–S. pneumoniae, SOFA and D-dimer-N. meningitidis. At multivariable analysis, the D-dimer has no effect on the whole population, but only on the subgroup of patients with N. meningitidis. As 500 ng/mL of D-dimer increase, the probability of a negative outcome increases on average by 4%*.

An acute change in total SOFA score ≥2 points, defining the sepsis-related organ dysfunction according to the sepsis-3 criteria, was present in 136 (84.0%) *S. pneumoniae* patients and in 86 (79.6%) *N. meningitidis* patients: the difference was not statistically significant.

### Invasive Infection Type

At the univariable and multivariable logistic regression analysis (Bayesian model averaging), the type of invasive infection (meningitis with BSI, meningitis without BSI, isolated BSI) was not found to be a variable significantly associated with in-hospital mortality (Table 2).

### Previous Splenectomy and Purpura at Admission

More patients with invasive infections due to *S. pneumoniae* than *N. meningitidis* previously underwent splenectomy (6.8 vs. 0.9%, *p* = 0.031), but at the univariable logistic regression analysis, a history of splenectomy was not found to be a variable significantly associated with in-hospital mortality (Table 2).

8.6% of *S. pneumoniae* patients had purpura at admission: 50% of these previously underwent splenectomy; out of the total of 11 splenectomized patients, 7 (63.6%) presented with purpura.

57.4% of *N. meningitidis* patients had purpura at admission: only 1 patient (1.6%) previously underwent splenectomy: in this patient D-dimer levels were <500 ng/mL.

Therefore, significantly more patients with invasive infections due to *N. meningitidis* compared to *S. pneumoniae* early presented purpura (57.4 vs. 8.6%, *p* < 0.001). Purpura was significantly more frequent in *N. meningitidis* than in *S. pneumoniae* patients regardless of D-dimer classes, and, independently by etiology, purpura prevalence was not significantly different between the different D-dimer classes. At the univariable logistic regression analysis (Table 2), the presence of purpura was not a variable significantly associated with in-hospital mortality.

### D-Dimer

Data analysis for continuous variables (Table 1) showed that D-dimer levels were significantly higher in *N. meningitidis* than *S. pneumoniae* patients: median 1,314 ng/mL (IQR 706–4,223) vs. 1,055 ng/mL (IQR 585–2,239), *p* = 0.009.

35/162 (21.6%) *S. pneumoniae* patients and 18/108 (16.7%) *N. meningitidis* patients had D-dimer <500 mg/dL.

101/162 (62.3%) of *S. pneumoniae* patients and 52/108 (48.1%) of *N. meningitidis* patients had D-dimer 500–3,000 ng/mL.

15/162 (9.3%) of *S. pneumoniae* patients and 15/108 (13.9%) *N. meningitidis* patients had D-dimer 3,000–7,000 ng/mL.

11/162 (6.8%) of *S. pneumoniae* patients and 23/108 (21.3%) *N. meningitidis* patients had D-dimer >7,000 ng/mL.

Most patients thus had D-dimer levels belonging to the class 500–3,000 ng/mL.

### Fibrinogen

Data analysis for continuous variables (Table 1) showed that fibrinogen levels were not significantly different between *S. pneumoniae* and *N. meningitidis* patients: median 619.5 mg/dL (IQR 469.0–777.0) vs. 574.5 mg/dL (IQR 427.0–720.0), *p* = 0.114.

At the logistic univariable regression analysis (Table 2) fibrinogen was not significantly associated with in-hospital mortality.

Only 4/249 (1.6%) patients, all presenting *N. meningitidis* BSI (1 isolated and 3 with meningitis), had fibrinogen levels <100 mg/dL; 3 of these 4 patients had shock and MODS, and 2 of these 3 died. Also elevating the cut-off to 200 mg/dL, we retrieved only 6 *N. meningitidis* and 3 *S. pneumoniae* patients with reduced levels: 7 of these 9 patients had shock and/or MODS and 4 out of these 7 patients ultimately died (mortality rate: 44.4%).

In definitive, only 9/249 patients (3.6%) had fibrinogen levels reduced under the normal value of 200 mg/dL.

### MODS

MODS occurred in 35.2% of *S. pneumoniae* patients and in 25.9% of *N. meningitidis* patients, without statistically significant difference (*p* = 0.101; Tables 1, **5**). No significant differences were observed within each single D-dimer class.

*Streptococcus pneumoniae* patients with D-dimer levels <500 and 500–3,000 ng/mL showed similar rates of early presentation of MODS (31.4 and 29.7%, respectively), and the percentages were about the same also comparing the classes 3,000–7,000 ng/mL and over 7,000 ng/mL (60 and 63.6%, respectively, **Table 5**); for D-dimer levels >3,000 ng/mL a statistically significant (*p* = 0.001) two-fold increase of occurrence of MODS was observed.

In *N. meningitidis* patients, the percentage of MODS progressively and significantly increased as D-dimer increased (**Table 5**), from 11.1% for values <500 ng/mL to 52.2% for values >7,000 ng/mL (*p* = 0.007); when D-dimer exceeded 7,000 ng/mL the rate of MODS significantly increased compared to lower values (*p* = 0.001).

Of the *S. pneumoniae* patients who died, 83.9% had MODS, while this percentage was only 23.7% among patients who survived: this difference was significant (*p* < 0.001). For *N. meningitidis* patients the corresponding percentages were 77.8 and 21.2%, and the difference was still significant (*p* = 0.001; Tables 3, 4). At the univariable logistic regression analysis, MODS was found to be a variable significantly (*p* < 0.001) associated with in-hospital mortality (Table 2).

**Table 3:** *Streptococcus pneumoniae*.

	**Survivors (*n* = 131)**	**Deaths (*n* = 31)**	***p*-value**
Males, *n* (%)	64 (48.9%)	15 (48.4%)	0.963
Septic shock, *n* (%)	22 (16.8%)	26 (83.9%)	**<0.001**
MODS, *n* (%)	31 (23.7%)	26 (83.9%)	**<0.001**
Purpura, *n* (%)	7 (5.3%)	7 (24.1%)	**0.004**
BSI isolated, *n* (%)	29 (22.1%)	1 (3.3%)	**0.020**
Meningitis isolated, *n* (%)	43 (32.8%)	3 (10.0%)	**0.013**
Meningitis + BSI, *n* (%)	59 (45.0%)	27 (87.1%)	**<0.001**
Fibrinogen (mg/dL), median (IQR)	621.0 (458.0–777.0)	590.0 (500.0–780.0)	0.766
D-dimer (ng/mL), median (IQR)	974.0 (513.0–1652.0)	1578.0 (944.0–2872.0)	**0.009**
Age, years, median (IQR)	47.0 (13.0–65.0)	63.0 (49.0–48.0)	**0.002**
SOFA, median (IQR)	3.0 (2.0–5.0)	7 (4.0–8.0)	**<0.001**
PLT (×10^9^), median (IQR)	200.0 (153.0–264.0)	169.0 (128.0–242.0)	0.085
ISTH score, median (IQR)	1.0 (1.0–2.0)	1.0 (1.0–3.0)	0.678
mISTH score, median [IQR]	1.0 [1.0 – 2.0]	1.0 [1.0 – 3.0]	0.550
SIC score, median (IQR)	3.0 (2.0–4.0)	3.5 (3.0–5.0)	**0.021**

*Association between variables and in-hospital mortality. Bold text indicates statistically significant difference (p < 0.05)*.

**Table 4:** *Neisseria meningitidis*.

	**Survivors (*n* = 99)**	**Deaths (*n* = 9)**	***p*-value**
Males, *n* (%)	55 (55.6%)	5 (55.6%)	1.000
Septic shock, *n* (%)	32 (32.3%)	8 (88.9%)	**0.001**
MODS, *n* (%)	21 (21.2%)	7 (77.8%)	**0.001**
Purpura, *n* (%)	57 (58.2%)	5 (62.5%)	1.000
BSI isolated, *n* (%)	16 (16.2%)	2 (22.2%)	0.643
Meningitis isolated, *n* (%)	23 (23.2%)	1 (11.1%)	0.680
Meningitis + BSI, *n* (%)	60 (60.6%)	6 (66.7%)	1.000
Fibrinogen (mg/dL), median (IQR)	576.0 (437.0–720.0)	233.0 (96.0–720.0)	0.233
D-dimer (ng/mL), median (IQR)	1140.0 (688.0–3543.0)	8051.0 (3859.0–40601.0)	0.001
Age, years, median (IQR)	15.0 (3.5–34.0)	5.0 (1.5–48.0)	0.739
SOFA, median (IQR)	3.0 (2.0–5.0)	6.5 (4.0–9.5)	**0.010**
PLT (×10^9^), median (IQR)	170.0 (129.0–232.0)	155.0 (131.0–175.0)	0.405
ISTH score, median (IQR)	2.0 (1.0–4.0)	5.0 (3.0–5.0)	**0.035**
mISTH score, median (IQR)	2.0 (1.0–4.0)	5.0 (3.0–5.0)	**0.041**
SIC score, median (IQR)	4.0 (3.0–4.0)	4.0 (3.5–5.0)	0.090

*Association between variables and in-hospital mortality. Bold text indicates statistically significant difference (p < 0.05)*.

### Septic Shock

The occurrence of septic shock was not significantly different between *S. pneumoniae* and *N. meningitidis* patients (29.6 vs. 37%, *p* = 0.229) (Table 1), and was very high regardless of D-dimer class (Table 5).

**Table 5:** Clinical status and outcomes in 270 patients with invasive bacterial infections by D-dimer class.

	***S. pneumoniae*** **(*****n*** **=** **162)**					***N. meningitidis*** **(*****n*** **=** **108)**				
**D-dimer class (ng/mL)**	**In-hospital mortality**	**Mortality + complications**	**MODS**	**Septic shock**	**Purpura**	**In-hospital mortality**	**Mortality + complications**	**MODS**	**Septic shock**	**Purpura**
<500	4/35 (11.4%)	12/35 (34.3%)^*^	11/35 (31.4%)	7/35 (20.0%)	2/35 (5.7%)^*^	0/18 (0%)	0/18 (0%)^*^	2/18 (11.1%)	6/18 (33.3%)	8/18 (44.4%)^*^
500–3,000	20/101 (19.8%)^*^	50/101 (49.5%)^*^	30/101 (29.7%)	29/101 (28.7%)	9/101 (9.0%)^*^	2/52 (3.8%)^*^	9/52 (17.3%)^*^	9/52 (17.3%)	17/52 (32.7%)	27/52 (51.9%)^*^
3,000–7,000	4/15 (26.7%)	6/15 (40.0%)	9/15 (60.0%)	7/15 (46.7%)	1/15 (7.1%)^*^	1/15 (6.7%)	3/15 (20.0%)	5/15 (33.3%)	5/15 (33.3%)	10/15 (66.7%)^*^
>7,000	3/11 (27.3%)	7/11 (63.6%)	7/11 (63.6%)	5/11 (45.5%)	2/11 (18.2%)^*^	6/23 (26.1%)	8/23 (34.8%)	12/23 (52.2%)	12/23 (52.2%)	17/23 (81.0%)^*^
Total	31/162 (19.1%)^*^	75/162 (46.3%)^*^	57/162 (35.2%)	48/162 (29.6%)	14/162 (8.7%)^*^	9/108 (8.3%)	20/108 (18.5%)	28/108 (25.9%)	40/108 (37.0%)	62/108 (57.4%)^*^

* *Statistically significant differences (p < 0.05) comparing S. pneumoniae vs. N. meningitidis*.

Of the *S. pneumoniae* patients who died, 83.9% had septic shock, while this percentage was only 16.8% among patients who survived: this difference was significant (*p* < 0.001). For *N. meningitidis* patients the corresponding percentages were 88.9 and 32.3%, and the difference was still significant (*p* = 0.001; Tables 3, 4). At the univariable logistic regression analysis, septic shock was found to be significantly (*p* < 0.001) associated with in-hospital mortality (Table 2).

About 33% of *N. meningitidis* patients with D-dimer levels <7,000 ng/mL and 52.2% of those with D-dimer levels >7,000 ng/mL early presented septic shock, without significant differences between every D-dimer classes (*p* = 0.411); also comparing only D-dimer classes under and over 7,000 ng/mL the difference was still not significant (*p* = 0.090).

### In-hospital Mortality (Primary Outcome)

In-hospital mortality was 14.8% (40/270 patients) for the overall population, and was significantly higher in *S. pneumoniae* than in *N. meningitidis* patients: 19.1 vs. 8.3%, respectively (*p* = 0.014; Table 1).

At the univariable logistic regression analysis, the type of invasive infection (meningitis with BSI, meningitis without BSI, isolated BSI) was not significantly associated with in-hospital mortality, and this data is conformed to the Bayesian model averaging (Table 2).

At the univariable logistic regression analysis (Table 2), ICU admission was found to be significantly associated with in-hospital mortality.

The median survival time in patients who died was 1 day (range 0–18 days) in the *N. meningitidis* group and 21 days (range 0–129 days) in the *S. pneumoniae* group.

At the analysis of the association between continuous variables and outcomes (Tables 3, 4), median D-dimer levels were significantly higher in non-survivors than in survivors, both for *S. pneumoniae* (1,578 vs. 974 ng/mL, *p* = 0.009) and *N. meningitidis* (8,051 vs. 1,140 ng/mL, *p* < 0.001).

In-hospital mortality gradually increased as D-dimer values increased, but the overall trend was significant for *N. meningitidis* group (*p* = 0.010) and non-significant for *S. pneumoniae* (*p* = 0.420).

At the univariable logistic regression analysis (Table 2), the following variables were significantly associated with in-hospital mortality: pneumococcal etiology (*p* < 0.001), female sex (*p* = 0.020), age (*p* < 0.001), ICU admission (*p* < 0.001), SOFA score (*p* < 0.001), septic shock (*p* < 0.001), MODS (*p* < 0.001), and D-dimer levels (*p* = 0.010).

For odds ratio (OR) of D-dimer, we considered an increase of 500 ng/mL. At multivariable analysis, the D-dimer did not show an effect on the whole population, but only on the group of *N. meningitidis* patients: in this group, as 500 ng/mL of D-dimer increased, the probability of unfavorable outcome increased on average by 4%.

For *N. meningitidis* the in-hospital mortality was 0% for D-dimer levels <500 ng/mL, very low (3.5%) for D-dimer levels <7,000 ng/mL, and increased to 26.1% for D-dimer exceeding the cut-off of 7,000 ng/mL (Table 5). Differences between classes were significant (*p* = 0.010), as well as between values under and above 7,000 ng/mL (*p* = 0.003).

**Figure 1:**
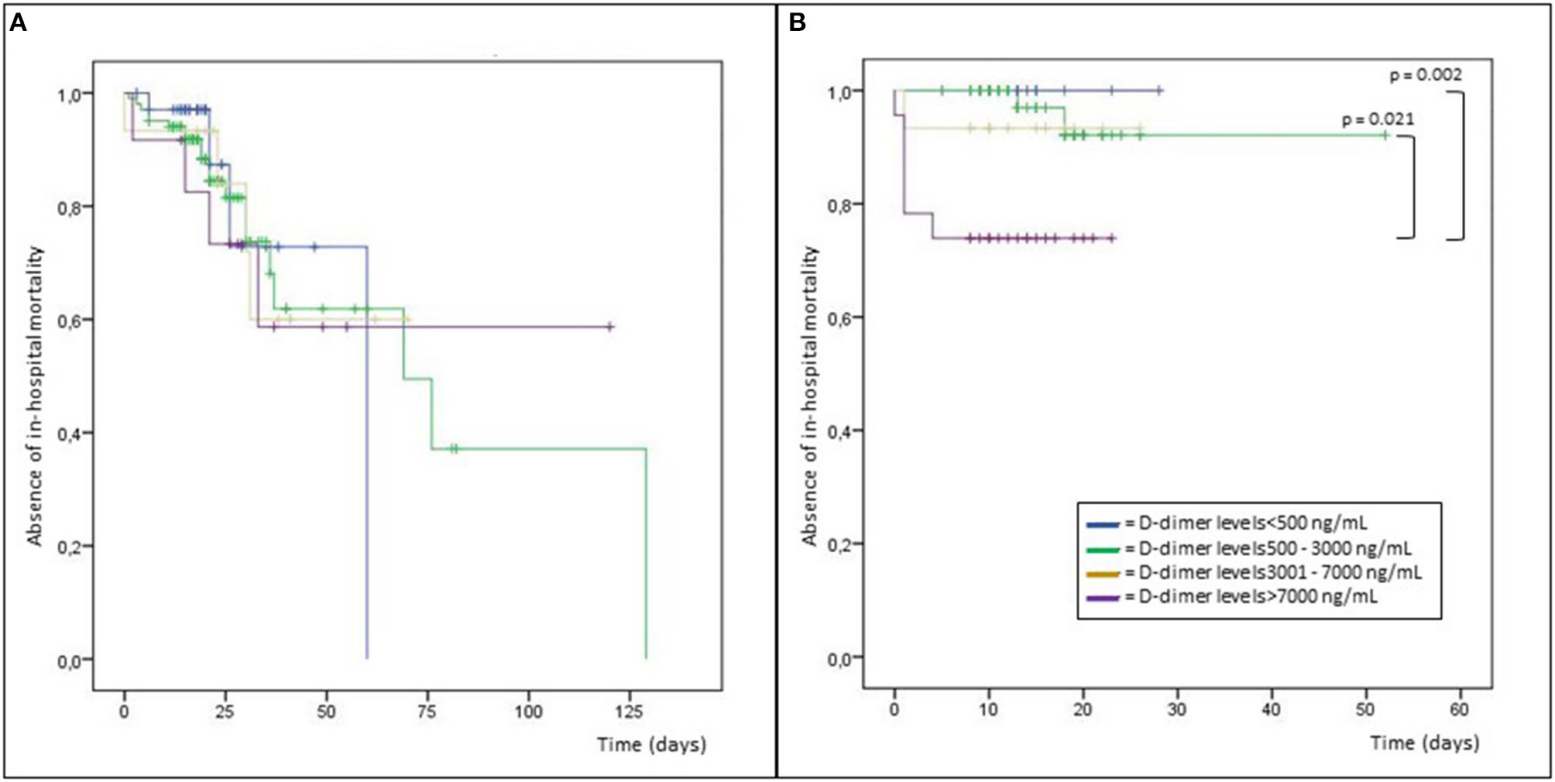
Kaplan-Meier analysis of in-hospital mortality in patients with infections due to *Streptococcus pneumoniae* **(A)** and *Neisseria meningitidis* **(B)**.

Kaplan-Meier analysis of in-hospital mortality showed for *N. meningitidis* subjects a statistically significant difference for D-dimer levels >7,000 ng/mL compared to values <500 ng/mL (*p* = 0.021) and to values 500–3,000 ng/mL (*p* = 0.002; Figure 1).

For *S. pneumoniae* the trend of the in-hospital mortality increasing as D-dimer increased was not statistically significant (*p* = 0.420), and the mortality was very high (11.4%) also in case of normal D-dimer levels <500 ng/mL (Table 5).

The mortality related to *S. pneumoniae* infections was always higher than *N. meningitidis* (Table 5), and was statistically significant in the D-dimer class of 500–3,000 ng/mL, representative of most population (19.8 vs. 3.8%, *p* = 0.008); for classes <500 and 3,000–7,000 ng/mL the overall trend was confirmed but without reaching the statistical significance (11.4 vs. 0%, *p* = 0.287, and 26.7 vs. 6.7%, *p* = 0.330), and for D-dimer values over 7,000 ng/mL the mortality was not different (27.3 vs. 26.1%, *p* = 1.000).

Among patients with purpura the in-hospital mortality was 50% in case of *S. pneumoniae* infections and 8.1% for *N. meningitidis*. Purpura did not correlate with in-hospital mortality in *N. meningitidis* patients (*p* = 1.000), but was significantly more frequent in non-survivors from invasive infections due to *S. pneumoniae* (24.1 vs. 5.3%, *p* = 0.004) (Tables 3, 4). At the univariable logistic regression analysis (Table 2), the presence of purpura was not significantly associated with in-hospital mortality.

Finally, we investigated the ability of D-dimer in predicting in-hospital mortality in the subgroup of patients early presenting with purpura. The rate of in-hospital mortality was 0% (0/45) in *N. meningitidis* patients with purpura and D-dimer levels <7,000 ng/mL and 29.4% (5/17) when D-dimer levels exceeded 7,000 ng/mL: this difference was statistically significant (*p* = 0.001).

The rate of in-hospital mortality was 0% (0/3) also in *S. pneumoniae* patients with purpura and D-dimer levels >3,000 ng/mL vs. 63.6% (7/11) among patients with D-dimer levels <3,000 ng/mL, but this difference was not significant (*p* = 0.192).

### Composite Outcome of In-hospital Mortality or Complications

The composite outcome of in-hospital mortality or complications was 35.2% (95/270 patients) for the overall population, occurring more frequently in patients with invasive infections due to *S. pneumoniae* than in those with *N. meningitidis* (46.3 vs. 18.5%, *p* < 0.001) (Table 1).

For D-dimer levels <500 and 500–3,000 ng/mL, this outcome occurred significantly more frequently in *S. pneumoniae* patients than those with infections due to *N. meningitidis* (34.3 vs. 0%, *p* = 0.004, and 49.5 vs. 17.3%, *p* < 0.001, respectively), while the trend was still evident but not statistically significant for classes 3,000–7,000 and >7,000 ng/mL (40 vs. 20%, *p* = 0.427, and 63.6 vs. 34.8%, *p* = 0.151, respectively) (Table 5).

Among *N. meningitidis* patients there were no complications when D-dimer values were <500 mg/dL, but the risk significantly increased for higher values (17.3% for 500–3,000 ng/mL, 20% for 3,000–7,000 ng/mL and 34.8% for >7,000 ng/mL, *p* = 0.029). In *S. pneumoniae* patients, this composite outcome occurred instead very frequently independently by D-dimer classes (ranging from 34.3 to 63.6%, *p* = 0.246) (Table 5).

In case of purpura, among *S. pneumoniae* patients this composite outcome was met in 10/14 patients (71.4%), in *N. meningitidis* patients in 19.4% (12/62).

Finally, we conducted a sub-analysis about the predictive ability of D-dimer in the subgroup of patients with purpura. This composite outcome occurred in 11.1% (5/45) of *N. meningitidis* patients with D-dimer levels <7,000 ng/mL and in 41.2% (7/17) in those with D-dimer levels >7,000 ng/mL: the difference was statistically significant (*p* = 0.013). In *S. pneumoniae* patients with D-dimer levels >3,000 ng/mL this outcome occurred in 33.3% (1/3), compared to 81.8% (9/11) in patients with D-dimer levels <3,000 ng/mL: this difference was not significant (*p* = 0.176).

## Discussion

Coagulopathy is crucially involved in the pathogenesis of sepsis-related dysregulated host response to infections: the differences between Gram-negative and Gram-positive bacteria, due to profound differences in their cell wall composition and the specificity of bacterial exotoxins production (18, 19), and the consequent interaction with the innate immune system, affect both the induction of immunothrombosis and the evolution of coagulopathy. The Pathogen-Associated Molecular Patterns (PAMPs) are indeed specific for different microorganisms and are basically involved in determining the coagulopathy-related organ damage: the lipopolysaccharide (LPS), one of the most important cell wall component of Gram-negative bacteria, is precociously recognized by innate immune system *via* toll-like receptor (TLR) 4, while the lipoteichoic acid, a cell wall component of Gram-positive bacteria, is mainly recognized by TLR2 (12); as differences in activation of these receptors can result in different production of inflammatory cytokines by the host, we should also expect a peculiar pattern of coagulopathy. During BSI due to *N. meningitidis*, besides the presence of elevated levels of circulating LPS, monocytes have been shown to express high level of functional TF, correlating with disease severity, and TF-bearing circulant MPs able to initiate the extrinsic coagulation pathway have been isolated in large amount (20). Moreover, human monocytes exposed to LPS produce both TF and plasminogen activator inhibitor (PAI)-2, both favoring fibrin deposition, but evidence showed that cellular production of these molecules may be uncoupled, since exposition to alloantigens leads to high levels of TF with no concomitant increase in PAI-2 activity (21). Hence, the procoagulant and the fibrinolytic pathways can be differently involved. Therefore, a great heterogeneity in the hemostatic aspects of the immune response and distinct patterns of coagulopathy in response to different microorganisms are expected, causing distinctive imbalance of the main determinants, namely the activation of coagulation, the down-regulation of physiological anticoagulants, and the inhibition of fibrinolysis (5).

Coagulation biomarkers could be useful to characterize the extent and/or type of coagulopathy occurring during sepsis and/or severe invasive infections due to different pathogens. In effect, thrombin-antithrombin complex, PAI-1 and D-dimer have been already proposed as potential biomarkers for the identification of the clinical phenotypes of sepsis, being significantly associated with the δ phenotype, which is characterized by a distinctive pattern of organ dysfunction and higher mortality (2); thereafter, such biomarkers might be useful in identifying those patients most likely to benefit from anti-inflammatory, anti-coagulant or immunomodulatory strategies (17).

Several coagulation factors could be measured, but screening all patients with an extensive panel of biomarkers would be expensive and dispersive; moreover, the complex balance between the pro- and anti-thrombotic and the pro- and anti-fibrinolytic drive occurring in response to different pathogens could be more simply synthetized by a molecule produced at the end of these interconnected cascades.

D-dimer satisfies several ideal criteria that a biomarker should have (3), such as the plausibility (the credible mechanism connecting the marker with the pathogenesis of the disease) and the coherence (the consistency of the association between the marker and the natural history of the disease). A biomarker should ideally be consistent, that is the association should persist in different individuals, in different places, in different circumstances, and at different times (3). Semeraro et al. (6, 7) reported a higher mortality in sepsis patients with low D-dimer, while other studies conducted on sepsis (10), but also on IE (8), BSI (9), and COVID-19 (11) conversely reported a worse prognosis for patients with high D-dimer values: in our opinion, these opposite findings should be interpreted not as an example of inconsistency of D-dimer as biomarker, but as the expression of its specificity to be differently associated with specific conditions and specific pathogens. This aspect has not been adequately taken into account in previous clinical studies: Schwameis et al. (9) enrolled in the same case series BSI due to *S. aureus, E.coli*, and *P. aeruginosa*, and Semeraro et al. (6) conducted their investigation using the data of the ALBIOS study (22), in which over 40% of patients did not have a microbiological diagnosis, and for the remaining no bacterial species was specifically reported or specifically studied for the interaction between coagulative biomarkers and clinical outcomes.

In our study we have clearly distinguished invasive infections due to *S. pneumoniae* from those due to *N. meningitidis*, and this was a strength of our study. Another strength is to have specifically investigated the association with relevant clinical outcomes depending by D-dimer levels early assessed at admission to the hospital. Median D-dimer levels were significantly higher in *N. meningitidis* than in *S. pneumoniae* patients.

Our results do not confirm the findings of Semeraro et al. (6, 7) about a higher mortality for low D-dimer levels, and agree with those of Turak et al. (8) and Schwameis et al. (9): we found for *N. meningitidis* an in-hospital mortality varying from 0% for D-dimer levels <500 ng/mL to 26.1% for values exceeding the cut-off of 7,000 ng/mL, progressively increasing on average by 4% as 500 ng/mL of D-dimer increase. For *S. pneumoniae* the mortality was always very high, regardless of D-dimer levels, being already 11.4% for normal values. Similarly, for *N. meningitidis* the composite outcome of mortality or complications increased as D-dimer increased. The strength of the association with the outcome (3), which is a central feature for a good biomarker, was thus more robust for invasive infections due to *N. meningitidis*. The only subgroup of our cohort in which lower D-dimer levels seemed associate with worse outcomes was that of *S. pneumoniae* patients with purpura, showing an in-hospital mortality of 63.6% when D-dimer levels were <3,000 ng/mL vs. 0% when D-dimer was >3,000 ng/mL: anyway, this difference was not significant. It is possible that the number of the sample size does not allow to highlight significant differences, so further studies are warranted to verify this finding.

It is noteworthy to observe that there have been no complications among *N. meningitidis* patients with normal D-dimer values <500 ng/mL, while for *S. pneumoniae* the composite of in-hospital mortality or complications occurred already in about one third of patients with normal values: this percentage was reached in *N. meningitidis* patients only for values >7,000 ng/mL. Therefore, D-dimer reflects only in part the complexity of the dysregulated host response finally leading to organ dysfunction and death. Since coagulopathy is a central element in the dysregulated host response leading to organ dysfunction, we assessed the association between D-dimer and MODS. In *N. meningitidis* infections the rate of MODS progressively and significantly increased as D-dimer increased; in *S. pneumoniae* invasive infections, a D-dimer cut-off of 3,000 ng/mL seems identify those patients with a significant two-fold increase of occurrence of MODS.

In our study, the mortality related to *S. pneumoniae* infections was higher than *N. meningitidis* in all D-dimer classes. A higher mortality in *purpura fulminans* due to *S. pneumoniae* compared to other pathogens (mainly *N. meningitidis*) has been recently reported by Contou et al. (23) in a multicenter French retrospective study including 306 cases admitted in ICUs from 2000 to 2016; the reason of a significantly higher mortality in that cohort compared to our data (41.2 vs. 14.8%) may be due to the different condition taken in account, specifically the enrollment only of the cases with evolution toward *purpura fulminans*. In our cohort only 8.6% of *S. pneumoniae* patients and 57.4% of *N. meningitidis* patients presented with purpura; like Contou et al. (23), also in our cohort the mortality was higher in this subgroup, 50 and 8.1% for *S. pneumoniae* and *N. meningitidis*, respectively, even if at the univariable logistic regression analysis purpura was not a variable significantly associated with in-hospital mortality.

The concepts of sepsis [according to the sepsis-3 definition (1)], coagulopathy (concerning pathophysiologic aspects and possibly reflected by D-dimer), overt-DIC [definable through clinical scores (13, 14)], even if interconnected, should be kept distinct, concerning different points of view of the same phenomenon, and it should be noted that these conditions often do not coexist, as evidenced by the discrepant percentages observed among our patients concerning presence of sepsis, elevated D-dimer levels, coagulopathy defined by SIC score (15), and overt-DIC defined by ISTH score (82.2, 80.4, 45.9, and 8.9%, respectively).

Finally, several studies have evaluated the role of prognostic biomarkers in case of bacterial meningitis: matrix metalloproteinases-8 assessed on CSF has been presented as an attractive prognostic biomarker in children (24), and longitudinal analysis of CSF lactate resulted to be an important predictor of prognosis (25). However, the advantage of measuring a biomarker directly from venous blood, in an easy and inexpensive way, with results rapidly available and everywhere, is evident.

D-dimer levels under a cut-off of 7,000 ng/mL, assessed within 24 h after admission, have shown to accurately predict a very low in-hospital mortality rate (3.5%) in case of infection due to *N. meningitidis*, and normal values <500 ng/mL excluded any further complications. For *S. pneumoniae*, D-dimer did not show the same ability to predict an increased risk of death, supporting the conclusion that D-dimer is a biomarker with a certain specificity in reflecting the dysregulated host coagulative response depending by specific pathogens.

### Study Limitations

The main limitation of this study is represented by the retrospective design, with bias and confounding being known errors potentially affecting the validity of these studies. Nevertheless, we conducted data analysis performing logistic regression analysis with Bayesian model averaging, to minimize confounders.

Another limitation is represented by a relatively small sample size that possibly did not allow to highlight significant differences in some cases, so further studies are warranted. Anyway, to the best of our knowledge, this cohort of patients with meningitis and/or BSI investigated for coagulation biomarkers early assessed within 24 h after admission represents one of the largest published so far.

Finally, our study has been conducted in a single center institution, thus composition of patient population, local resources, medical protocols, and staffing characteristics may limit the generalizability of results.

## Conclusions

D-dimer is rapid to be obtained, at low cost and available everywhere, and seems to be an interesting biomarker able to reflect the concept of the dysregulated host response in case of invasive infections due to *N. meningitidis* and *S. pneumoniae*.

Assessment of D-dimer within 24 h after admission can help stratify the risk of in-hospital mortality and complications in patients with meningitis and/or BSI due to *N. meningitidis*: D-dimer values <500 ng/mL exclude any further complications, and a D-dimer cut-off of 7,000 ng/mL seems able to predict a significantly increased risk of in-hospital mortality from much <10% to over 25%. For *S. pneumoniae* invasive infections the mortality risk resulted always high, over 10%, irrespective by D-dimer values.

## Data Availability Statement

The original contributions presented in the study are included in the article/supplementary material, further inquiries can be directed to the corresponding author.

## Ethics Statement

Ethical review and approval was not required for the study on human participants in accordance with the local legislation and institutional requirements. Written informed consent for participation was not provided by the participants' legal guardians/next of kin because for the retrospective nature of the study.

## Author Contributions

All authors made substantial contributions to the conception of the work, acquisition, interpretation of data, in drafting the work, and approved the submitted version.

## Conflict of Interest

CT has received funds for speaking at symposia organized on behalf of Pfizer, Novartis, Merck, Angelini, Zambon, Thermofischer, Biotest, Gilead, Hikma, Biomerieux, and Astellas. The remaining authors declare that the research was conducted in the absence of any commercial or financial relationships that could be construed as a potential conflict of interest.
